# Deep Learning in Cardiothoracic Ratio Calculation and Cardiomegaly Detection

**DOI:** 10.3390/jcm13144180

**Published:** 2024-07-17

**Authors:** Jakub Kufel, Iga Paszkiewicz, Szymon Kocot, Anna Lis, Piotr Dudek, Łukasz Czogalik, Michał Janik, Katarzyna Bargieł-Łączek, Wiktoria Bartnikowska, Maciej Koźlik, Maciej Cebula, Katarzyna Gruszczyńska, Zbigniew Nawrat

**Affiliations:** 1 Department of Radiology and Nuclear Medicine, Faculty of Medical Sciences in Katowice, Medical University of Silesia, Medyków 14, 40-752 Katowice, Poland; 2 Tytus Chalubinski’s Hospital in Zakopane, 34-500 Zakopane, Poland; 3 Bright Coders’ Factory, Technologiczna 2, 45-837 Opole, Poland; 4 Faculty of Medicine in Katowice, Medical University of Silesia, Medyków 18, 40-752 Katowice, Poland; 5 Students’ Scientific Association of Computer Analysis and Artificial Intelligence, Department of Radiology and Nuclear Medicine, Medical University of Silesia, Medyków 14, 40-752 Katowice, Poland; 6 Paediatric Radiology Students’ Scientific Association, Division of Diagnostic Imaging, Department of Radiology and Nuclear Medicine, Faculty of Medical Science in Katowice, Medical University of Silesia, 40-752 Katowice, Poland; 7 Division of Cardiology and Structural Heart Disease, Medical University of Silesia, 40-635 Katowice, Poland; 8 Individual Specialist Medical Practice Maciej Cebula, 40-239 Katowice, Poland; 9 Foundation of Cardiac Surgery Development, 41-800 Zabrze, Poland; 10 Department of Biophysics, Faculty of Medical Sciences in Zabrze, Medical University of Silesia, 41-808 Zabrze, Poland

**Keywords:** chest radiograph, cardiothoracic ratio, cardiomegaly, convolutional neural network, segmentation

## Abstract

**Objectives**: The purpose of this study is to evaluate the performance of our deep learning algorithm in calculating cardiothoracic ratio (CTR) and thus in the assessment of cardiomegaly or pericardial effusion occurrences on chest radiography (CXR). **Methods**: From a database of 8000 CXRs, 13 folders with a comparable number of images were created. Then, 1020 images were chosen randomly, in proportion to the number of images in each folder. Afterward, CTR was calculated using RadiAnt Digital Imaging and Communications in Medicine (DICOM) Viewer software (2023.1). Next, heart and lung anatomical areas were marked in 3D Slicer. From these data, we trained an AI model which segmented heart and lung anatomy and determined the CTR value. **Results**: Our model achieved an Intersection over Union metric of 88.28% for the augmented training subset and 83.06% for the validation subset. F1-score for subsets were accordingly 90.22% and 90.67%. In the comparative analysis of artificial intelligence (AI) vs. humans, significantly lower transverse thoracic diameter (TTD) (*p* < 0.001), transverse cardiac diameter (TCD) (*p* < 0.001), and CTR (*p* < 0.001) values obtained using the neural network were observed. **Conclusions**: Results confirm that there is a significant correlation between the measurements made by human observers and the neural network. After validation in clinical conditions, our method may be used as a screening test or advisory tool when a specialist is not available, especially on Intensive Care Units (ICUs) or Emergency Departments (ERs) where time plays a key role.

## 1. Introduction

Chest radiography (CXR) is one of the most frequently performed imaging procedures in clinical diagnosis, representing 40% of the 3.6 billion imaging procedures performed worldwide every year [1]. The National Health Service (NHS) of the United Kingdom (UK) reports that around 2.2 million CXRs are ordered by general practitioners performed each year [2]. According to the Federal Office for Radiation Protection in Germany, CXR accounted for about 13 million X-ray examinations in 2018 [3]. Its main advantage is high availability (regardless of the size and location of the medical facility), low cost, and wide diagnostic use in many diseases, including lung and heart diseases. One useful measurement determined on CXR is the cardiothoracic ratio (CTR), which is the ratio of the width of the heart silhouette (TCD—transverse cardiac diameter) to the widest dimension of the chest (TTD—transverse thoracic diameter). CTR helps in detecting enlargement of the heart silhouette, which usually indicates cardiomegaly, but can also be the result of pericardial effusion [4]. A value greater than 0.50 (normal: 0.42–0.50) in an adult who is considered pathological [5]. Although the diagnostic and predictive value of this parameter is losing importance in relation to modern methods such as echocardiography of determining the size of the heart, CXR and CTR are still valuable in the assessment of heart size in the hospital emergency room (ER) or intensive care unit (ICU) [6].

According to literature reports, the CTR should be determined in the posteroanterior (PA) projection [7,8], even though there are also studies containing appropriate values for CTR in the anteroposterior (AP) projection [5]. The cut-off value for CTR may vary, depending on the projection of the study and the group of patients in whom the study is performed. The PA projection is preferred because the silhouette of the heart is closest to its true dimensions. In the case of the AP projection, the heart is closer to the cassette, which makes its silhouette enlarged. Even so, AP is indispensable in the diagnosis of bedridden patients and small children.

Cardiomegaly is a broad term for various conditions that cause the heart’s enlargement, often going undiagnosed until symptoms appear. It affects nearly 5.8 million people in the United States. There are many reasons for the development of cardiomegaly—from coronary artery disease and myocardial infarction, through valvular regurgitation, various cardiomyopathies, to physiological conditions such as the athlete’s heart or pregnancy [9]. Many of these are pathological conditions, which may lead to heart failure, resulting in up to a 50% five-year survival rate [10]. Considering the potential risk underlying this pathology, CTR should be assessed in each chest X-ray examination. However, it is a time-consuming process. Artificial intelligence (AI) and its subset, deep learning (DL), have proven to be helpful in improving the effectiveness of diagnostic imaging in medicine. Thanks to the use of convolutional neural networks (CNNs), the diagnosis of pathology on CXR has reached a level comparable to the effectiveness of trained radiologists [11,12].

This article focuses on the possibility of more effective usage of deep learning in clinical diagnostics. To conduct the study, the standardized digital imaging and communications in medicine (DICOM) format was used, which enables efficient distribution of datasets. The aim was to evaluate the performance of our deep learning algorithm in calculating CTR, and thus predict the possibility of such diseases as cardiomegaly or pericardial effusion on chest X-rays. The algorithm was compared with physicians, whose assessment was used as a reference method. The correctness of anatomical segmentation of the heart and lungs performed by physicians and artificial intelligence (AI) was also analyzed.

## 2. Materials and Methods

### 2.1. Radiological Phase

To ensure the highest possible standard of this study, a preliminary analysis and selection of images was carried out from a database of about 8000 anonymized CXRs. The patients from whom the images were taken during their admission to the clinical hospital gave their consent to the collection and processing of their data. The exclusion criteria for CXR images were as follows: images were not of the chest; images with an abnormal range not covering all chest structures; rotated images; incorrectly exposed images; images with movement artifacts; and images of children.

The entire dataset was divided into 13 folders containing a comparable number of CXRs. In total, 4 investigators (3 doctor interns and 1 radiology resident doctor) pre-analyzed the contents of the folders to determine the projection in which the images were taken: standing (PA), or prone (AP). A total of 1020 CXRs were randomly selected from all analyzed images proportionally from each of the 13 folders. Because CXR images of both PA and AP were included in this study, different CTR threshold values were set depending on the position of the patient: >0.55 for the standing position, >0.58 for the supine position, based on previous studies [5,13,14,15].

Afterward, the same four investigators independently calculated the CTR by measuring the width of the heart and lungs separately. The CTR was calculated for all eligible studies using the freely available RadiAnt DICOM Viewer software (2023.1). The chest width was measured at the widest point, usually at the height of the diaphragm domes. Heart width measurements were made with respect to a vertical line drawn along the spinous processes of the vertebrae.

**Figure 1:**
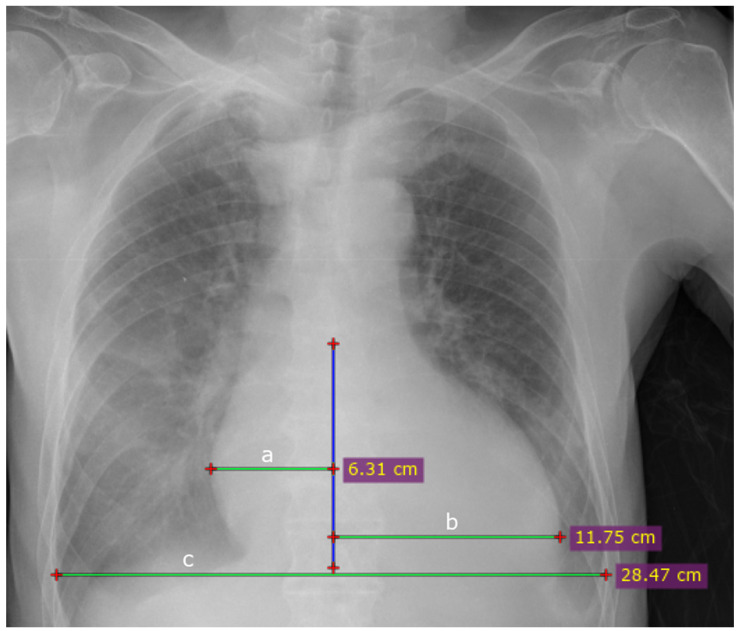
How measurements were manually determined using RadiAnt: blue vertical line—a line drawn through the spinous processes of the thoracic spine vertebrae; a—right side of the heart; b—left side of the heart; a + b = the widest dimension of the heart; c—the widest dimension of the chest.

An orthogonal line segment was then drawn from the vertical line to the farthest border of the heart separately on the left and right sides. The sum of the left and right segments determined the measurement of the width of the heart silhouette (Figure 1). During CTR calculations, the time needed to estimate TCD and TTD was measured each time for all four researchers. A table containing the individual results of the measurements (TCD, TTD, CTR, measurement time) made by the researchers and AI can be found in the Supplementary Materials (Table S1).

**Figure 2:**
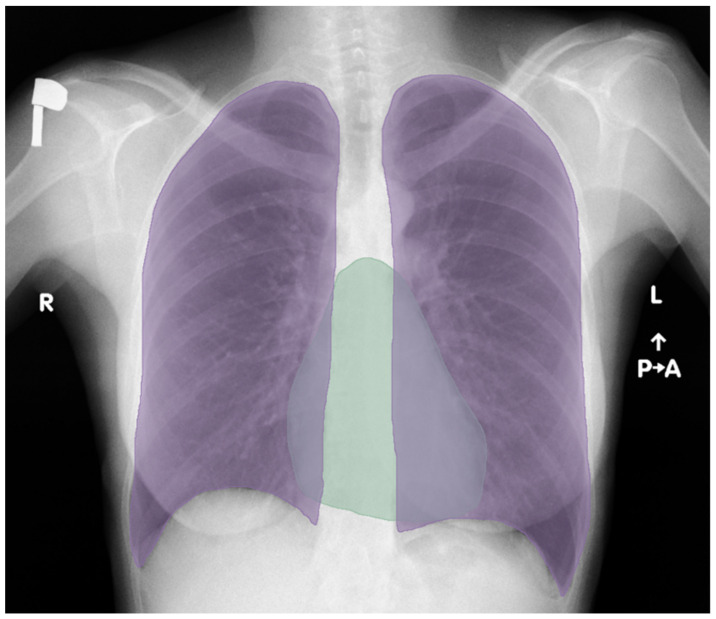
How annotations were applied to the anatomical areas of the heart (green) and lungs (purple).

Then, the four investigators independently marked a total of 1059 CXR anatomical areas of the heart and lungs in the 3D Slicer program, which is used to make annotations on radiological images. The masks were used in subsequent stages to train the artificial neural network model. The finished markings were re-checked by another two independent researchers with the most experience to eliminate potential errors. Both lungs were marked with the same color as mask #1. The lung mask covered the area from the inner surfaces of the ribs, including the shadow of the mediastinum, bilaterally to the paraspinal line. The heart has been marked with a separate color as mask #2 (Figure 2), excluding trunks of large venous and arterial vessels. It was decided to overlap the masks in the areas where the shadows of the heart overlap with the areas of the lungs to increase the accuracy of measurements and receive the most accurate mapping of anatomical structures.

### 2.2. Technical Materials

To conduct machine learning experiments in the radiological domain, usually non-standard software is required to combat such aspects as overlapping annotations, annotation management, annotation storage, versioning, and verification of resulting segmentations.

### 2.3. Slicer and Custom Workflow

We decided to use industry standard medical annotation software, 3D Slicer (5.1.0-2022-11-16) [16]. It supports DICOM files [17] as well as standard non-overlapping segmentations and plugins which can be developed in the Python 3.10 programming language [18].

The plugin was developed to improve and enable multi-class overlapping segmentation with minimal effort from the radiological team.

The label list can be updated from a remote server and users can select, visualize, annotate, load, and save segmentation classes. To standardize the format for saving segmentations in a replicable and robust way, the zarr library [19] was used. Annotations were uploaded to the Google Drive cloud, where each annotator had their own folder in which the annotations were saved. This allowed for rapid collaboration between team members of different specializations.

### 2.4. Data Preparation and Dataset

We sourced about 8000 images in total, out of which 1059 were segmented with the heart and lungs label and 4072 had their CTR measured, which we used for comparison. Segmented images were split into two subsets: training (80%—847 images), used for fitting the U-Net model; and validation (20%—212 images) used for monitoring and validation of the training process. Image preparation consisted of the following steps:

1. Inversion of values of Monochrome1 to Monochrome2 images.
2. Scaling to Hounsfield units (HU).
3. Downsampling to 256 × 256.
4. Standardization to zero mean and unit variance (computed with training dataset).

In order to increase robustness of the model, the training data were augmented by randomly flipping images vertically and horizontally, as well as rotating by −0.2:+0.2 radians and −20:+20% horizontal and vertical random shift.

### 2.5. Semantic Segmentation—U-Net

Semantic segmentation is a supervised task in which each pixel has a class label assigned to it according to a predicted object type. It usually forms clusters which can be grouped into objects. One of the models which has been successfully applied to segmentation tasks, especially medical segmentation, is U-Net [20]. U-Net combines down/up-sampling paths and skip connections, with activation maps from different stages of forward propagation. The resolution of activation maps decreases with the combination of convolutional and pooling layers until the middle of the network and then is gradually increased by transposed convolution layers.

### 2.6. Model—Training Details

U-net can be adjusted to one’s hardware and time resources by adjusting the number of layers and filters. We have trained a 32-layer neural network with 1.94 M parameters. An Adam optimizer with learning rate 0.001 with lr reduction on plateau was used. The network was trained for 56 epochs. A combination of focal and dice loss was used as a loss function. Using Google Colab Pro (Tesla A100) training took 9.33 min total, with each step with a batch size of 64 taking 0.8 s.

### 2.7. Postprocessing and CTR Calculation

CTR was computed with a non-AI algorithm, utilizing standard computer vision operations. The goal was not only to compute the width of the object(s), but also eliminate any remaining artifacts that may be the result of AI-based segmentation.

## 3. Results

A total of 1059 hearts and 2118 lungs were segmented on 1059 chest radiographs. 

**Figure 3:**
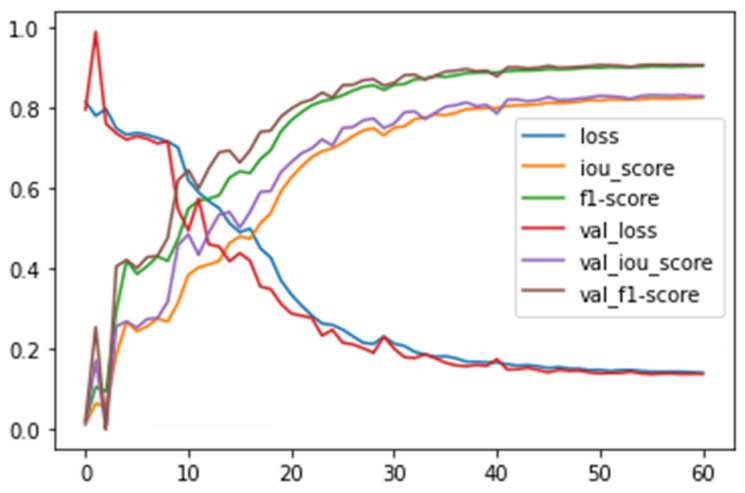
Metrics, loss, and learning rate during neural network training.

Metrics were observed during and at the end of training for both augmented training and validation subsets. Results were saved after each completed epoch. The model achieved an Intersection over Union (IoU) metric of 88.28% for the augmented training subset and 83.06% for the validation subset. F1 scores for each subset were 90.22% and 90.67%. Higher validation metrics were recorded due to data augmentation technique, which regularizes the training-reducing factor model of previously seen data (Figure 3). 

We also measured the average inference time (1.839 ms; P100 GPU) while total time, including segmentation post-processing and CTR calculation (4.706 ms).

A Lilliefors test (*p* < 0.01) and Kolmogorov–Smirnov test (*p* < 0.05) were used to assess the normality of the distribution of quantitative variables.

### 3.1. Comparative Analysis AI vs. Humans

The average combined measurement time for TCD, TTD, and CRT in the entire dataset was 17.00 s for the researchers and 0.004706 s for AI.

**Figure 4:**
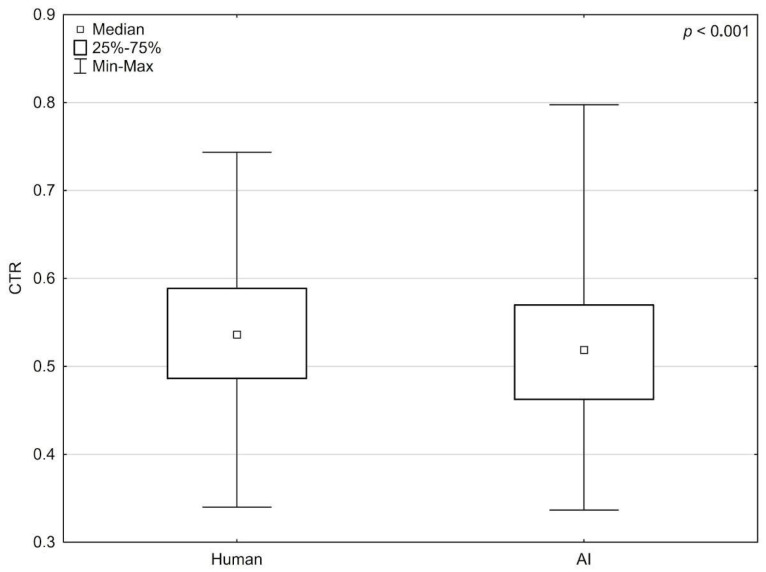
Difference in mean obtained by the humans observers and AI in the cardiothoracic ratio.

A U-Mann–Whitney test was used to assess the differences between the observers and the neural network. The results of all four observers were averaged. Extremes and outliers were discarded before comparison. Significantly lower TTD (*p* < 0.001), TCD (*p* < 0.001), and CTR (*p* < 0.001) values obtained using the neural network were observed (Figure 4).

### 3.2. Comparative Analysis of the Observer vs. Observer

**Figure 5:**
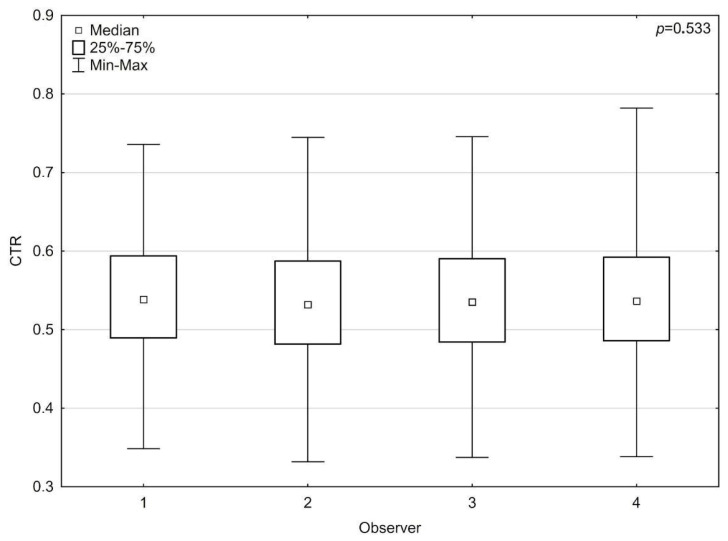
Difference between the human observers in the cardiothoracic ratio.

A Kruskal–Wallis test was used to assess differences between observers. There were no significant differences in TTD (*p* = 0.823), TCD (*p* = 0.455), and CTR (*p* = 0.533) measurements (Figure 5). 

### 3.3. Correlation Strength Analysis (CTR_AI vs. CTR_Human)

**Figure 6:**
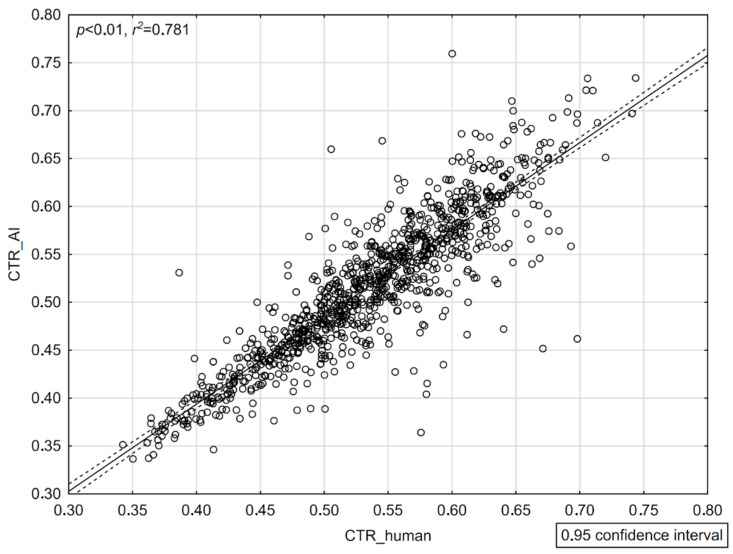
Correlation of the mean obtained by the human observers and AI for the cardiothoracic ratio.

To assess the strength of the correlation between the observers and the neural network, a Spearman rank correlation test was used, obtaining significant, positive, and strong correlations between TTD (*p* < 0.01, r^2^ = 0.875), TCD (*p* < 0.01, r^2^ = 0.815), and CTR (*p* < 0.01, r^2^ = 0.781) (Figure 6).

## 4. Discussion

The CTR derived from CXR is an important tool for the assessment of heart diseases, such as cardiomegaly and pleural effusion [21,22]. However, because it requires manual measurement, is time consuming and is subject to error. The method of automatic determination of CTR has been technically validated in several studies [23,24]. However, there are few studies with clinical evaluation of this solution. Saiviroonporn et al., which validated manual CTR measurements and measurements performed with the use of artificial intelligence, rated AI assistance in diagnosing cardiomegaly at 40% as excellent, 56% as good and 4% as poor [25]. The authors of Arsalam et al. point out the time-consuming nature and the need to involve experienced medical experts in performing CTR measurements, who could be replaced by artificial intelligence and point out that cardiomegaly and related diseases have been successfully diagnosed with the use of artificial intelligence [26]. Ajmera et al. showed that the artificial intelligence model they used for CTR measurements not only achieved high specificity (>99%) and sensitivity (80%), but also contributed to increasing the efficiency of the radiologist assessing X-ray images. However, these studies concerned the comparison of CXR mainly unchanged and only in the standing position in the PA projection [27].

In our work, a broader dataset was used to train and validate a deep learning model. We are the first to include CXR in both PA and AP projections, which reflects the reality of working in a hospital with patients in various conditions. We are also the first to use a segmentation method in which anatomical areas overlap, which contributes to a better representation of these structures. All CXRs came from a hospital database, which contained many diseases and artifacts, such as drains, cables, and clothing. Our dataset thus represents conditions found in real clinical practice.

The trained model, based on the determinations carried out by our doctors, performed segmentation of the heart and lungs which were later used to calculate the CTR. The test set demonstrated high efficiency and accuracy of deep learning model segmentation, supported by qualitative (IoU, F1) and quantitative methods, comparing them with expert segmentations. The CTR values derived from the model correlated significantly with the average of the values obtained by four independent observers. The visible significant difference between the observers and AI in terms of the obtained CTR values, as well as TCD and TTD, suggests that the model underevaluates measurement values. In the retrospective analysis of some significantly underevaluated cases, it was noted that the discrepancies between the determined TCD and TTD resulted from significant scoliosis, asymmetry in the AP projection, or consolidation in the lung field projection. These factors did not significantly affect the values obtained by four independent observers, as the results of their measurements (CTR, TCD, TTD) did not significantly differ from each other. In our analysis, we decided to include studies that are difficult to assess due to our desire to reproduce the real work environment, realizing that this will affect the results. Therefore, CXR in the AP projection in bedridden patients, including ICU patients, was included in the study group.

The advantage of our tool is the fact that it allows you to shorten the interpretation time of CXR (17.00 s for researchers, 0.004706 s for AI). The radiologist usually must manually mark the segments on the image and calculate the index using the formula. Our tool takes only a few milliseconds to calculate CTR, freeing the radiologist to focus on interpreting other pathologies. Ajmera et al. obtained a CXR interpretation time of 2 s for the DL model they described [27]. Saiviroonporn et al. showed that the use of AI even as a supporting tool during manual determination of CTR values speeds up the performance of measurements almost five times (manual method—t 10.6 ± 1.5 s per case vs. manual + AI 2.2 ± 2.4 s) [25].

The application of AI in medical visualization, focusing on its role in medical imaging and image processing. AI, in particular machine learning and deep learning techniques, are being used to improve image quality, automate analysis, and support diagnosis. With AI, it is possible to detect subtle pathological changes, which contributes to early diagnosis and more precise treatment. In addition, AI plays a key role in the personalization of healthcare, providing doctors with advanced tools to interpret images and make clinical decisions [28].

## 5. Conclusions

To improve our model, it is necessary to train the neural network model on a larger amount of data. Moreover, CXRs should be used both in AP and PA positions with various pathologies so that they best reflect the working conditions in the hospital. In the future, after validation in clinical conditions, it will be able to provide an advisory tool for doctors to improve their work and act as a quick screening test when a specialist doctor is not available. Use can be made during chest screening, e.g., periodic examinations of employees or admission to a new job. The algorithm could have a significant impact on shortening the time of the diagnostic process (by three orders of magnitude), as well as extending CXR examination descriptions by automatically marked CTR each time. During this time, the physician describing the image could focus on the analysis of other pathologies visible in the examination. The results presented in this paper are very promising and confirm that there is a significant, positive, and strong correlation between the measurement results marked by the observers and the neural network model, which proves the great potential of using our tool to work in clinical conditions.

## Data Availability

The original contributions presented in the study are included in the article/Supplementary Material; further inquiries can be directed to the corresponding author/s.

## Supplementary Materials

The following supporting information can be downloaded at: https://www.mdpi.com/article/10.3390/jcm13144180/s1. Table S1: Measurements.
